# Uptake of Vitamins D_2_, D_3_, D_4_, D_5_, D_6_, and D_7_ Solubilized in Mixed Micelles by Human Intestinal Cells, Caco-2, an Enhancing Effect of Lysophosphatidylcholine on the Cellular Uptake, and Estimation of Vitamins D’ Biological Activities

**DOI:** 10.3390/nu13041126

**Published:** 2021-03-29

**Authors:** Eiichi Kotake-Nara, Shiro Komba, Megumi Hase

**Affiliations:** Food Research Institute, National Agriculture and Food Research Organization, 2-1-12 Kannondai, Tsukuba 305-8642, Ibaraki, Japan; skomba@affrc.go.jp (S.K.); meguhase@icloud.com (M.H.)

**Keywords:** Caco-2 cells, intercellular barrier formation, intestinal uptake, lysophosphatidylcholine, PASS, mixed micelles, vitamin D

## Abstract

Vitamins D have various biological activities, as well as intestinal calcium absorption. There has been recent concern about insufficient vitamin D intake. In addition to vitamins D_2_ and D_3_, there are lesser-known vitamins D_4_–D_7_. We synthesized vitamins D_5_–D_7_, which are not commercially available, and then evaluated and compared the mixed micelles-solubilized vitamins D uptake by Caco-2 cells. Except for vitamin D_5_, the uptake amounts of vitamins D_4_–D_7_ by differentiated Caco-2 cells were similar to those of vitamins D_2_ and D_3_. The facilitative diffusion rate in the ezetimibe inhibited pathway was approximately 20% for each vitamin D type, suggesting that they would pass through the pathway at a similar rate. Lysophosphatidylcholine enhanced each vitamin D uptake by approximately 2.5-fold. Lysophosphatidylcholine showed an enhancing effect on vitamin D uptake by reducing the intercellular barrier formation of Caco-2 cells by reducing cellular cholesterol, suggesting that increasing the uptakes of vitamins D and/or co-ingesting them with lysophosphatidylcholine, would improve vitamin D insufficiency. The various biological activities in the activated form of vitamins D_4_–D_7_ were estimated by Prediction of Activity Spectra for Substances (PASS) online simulation. These may have some biological activities, supporting the potential as nutritional components.

## 1. Introduction

Vitamin D is required for intestinal absorption of calcium and phosphorus and has an important role in maintaining homeostasis of bone. Vitamin D deficiency causes rickets for children and osteomalacia for adults. In the elderly, vitamin D is an important nutritional component for prevention of osteoporosis, frailty, and locomotive syndrome.

Geographical and seasonal factors are well known to limit the biosynthesis of vitamin D_3_ by sun exposure. Especially in high latitudes > 35, the irradiation intensity of sunlight UV is weak, which is thought to reduce biosynthesis of vitamin D_3_ to insufficient quantities [1]. In addition, sunscreen with a high sun protection factor significantly inhibits vitamin D_3_ biosynthesis regardless of latitude, even in summer [2]. Aging also causes a decrease in the ability to produce 7-dehydrocholesterol, a vitamin D_3_ precursor [3]. Conversely, sunlight also has risks to the appearance and health of humans. UV reportedly can induce skin cancer [4], wrinkle formation [5,6,7], macular degeneration of the retina, and cataracts [8], suggesting that a vitamin D supply that is too dependent on sunlight may cause vitamin D deficiency due to habits, lifestyle, geography, and aging.

On the other hand, vitamin D is found in some foods. The anti-rickets component in cod liver oil was revealed to be vitamin D_3_ in 1936 [9]. However, fish oil has a flavor and odor problem, so direct intake has not generally been preferred. Other than cod liver oil, rats studies in the 1920s showed that coconut oil [10], alfalfa, and clover [11] contained an anti-rickets component, but the identity of the component was not unknown. UV irradiation has an anti-rickets effect not only in humans and animals, but also in foods. Although wheat, lettuce, cottonseed oil, linseed oil [12], yeast [13], cow and goat milk [14], lard, olive oil [15], and malt [16] did not exhibit anti-rickets activity by themselves, exposure of these foods to UV irradiation produced anti-rickets activity, but vitamin D had not yet been discovered at the time of these studies. These foods were thought to contain some precursors that were converted to vitamin D by UV. In 1926, ergosterol contained in mushrooms or fungi was found to be a precursor of vitamin D_2_ [17].

**Figure 1:**
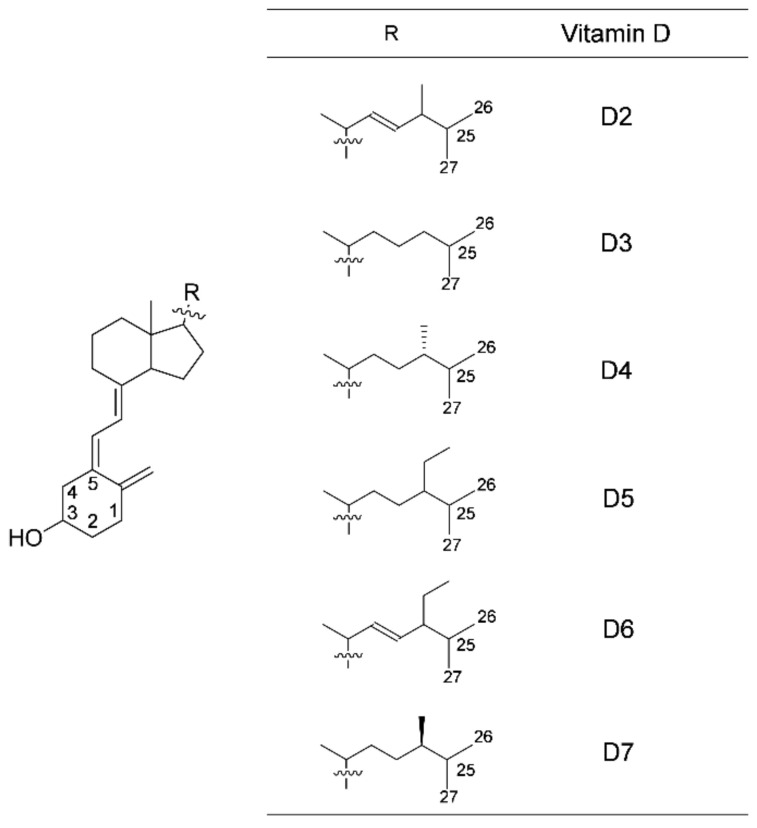
Chemical structures of vitamins D. The numbers in the structural formula indicate the locations of the carbons.

Even before the isolation of vitamin D_2_, fortified foods containing anti-rickets factor (vitamin D_2_) produced by UV irradiation to foods were developed and helped prevent onset of the disease [18]. However, foods that naturally contain vitamin D are very limited among readily available foods and at present include fish, shellfish, mushrooms, and eggs. Since vitamin D is usually not found in fruits and vegetables, for vegans and vegetarians and even people who eat meat and seafood, their eating habits may not ingest enough vitamin D from their diets. In fact, a large-scale cohort study showed that 81.3% of Japanese were vitamin D deficient [19]. In mothers, it has been found to affect subsequent fetal development [20,21]. Therefore, it is important to keep vitamin D levels inside the body by somehow continuously ingesting vitamin D from foods. We believe that this can be achieved by two methods: (i) increasing the intake of diverse foods containing vitamin D and (ii) enhancing the intestinal absorption of vitamin D (in other words, improved intestinal absorption even with few intake opportunities).

(i): In addition to vitamins D_2_ and D_3_, there are vitamins D_4_–D_7_, although they are not well known (Figure 1). There are many unclear points about what kinds of foods contain these forms and in what amounts. Ingestion of various types of vitamin D from diverse foods may lead to increased opportunities for intake of vitamin D. Vitamin D has poor bioavailability as do other fat-soluble components, such as carotenoids. After ingesting foods with certain compounds, such as carotenoids, vitamin D is also considered to be released from the matrix of food in the digestive tract and incorporated into an emulsion dispersed with bile and fats derived from the food. By pancreatic juice action, vitamin D is solubilized in mixed bile salt micelles that are absorbed in the intestinal tract. We have previously studied the intestinal absorption of carotenoids [22,23,24,25,26] and, here, have tried to apply a similar method to the study of intestinal absorption of vitamin D. Unfortunately, vitamins D_5_–D_7_ are not commercially available. Therefore, we have recently obtained these vitamins D by organic synthesis from commercial plant sterols as starting materials via a 7-dehydro form [27]. In the present study, to investigate the intestinal absorption of six types of vitamin D, which were commercially available vitamins D_2_–D_4_ and organically synthesized vitamins D_5_–D_7_, we evaluated and compared the uptake of vitamin D solubilized in mixed micelles composed of taurocholate, monoacylglycerol, fatty acid, and phospholipid by intestinal model Caco-2 cells.

(ii): Enhancing the intestinal absorption of vitamin D that is, adopting an efficient absorption method, even if the intake opportunity is small, would help prevent vitamin D deficiency. Our group has previously evaluated 28 kinds of polar lipids, such as glycerophospholipids and glyceroglycolipids, involved in the uptake and/or secretion of carotenoids [22,23,24,25,28]. Among the 28 kinds, 1-16:0-2-OH-*sn*-glycerol-3-phosphocholine (lysophosphatidylcholine) showed the strongest enhancing effect. We have also revealed that cell-cell adhesion/cell-matrix adhesion was involved in the mechanism of the enhancing effect [24,25]. In other words, uptake and secretion were enhanced by the influence of lysophosphatidylcholine on cells other than mixed micelles. This finding suggests that the enhancing effect of lysophosphatidylcholine also can be applied to fat-soluble components other than carotenoids. In the present study, we also determined if lysophosphatidylcholine raises the uptake of vitamin D by Caco-2 cells. Regarding the enhancing effect mechanism, we previously predicted that lysoglycerolipids decreased cellular cholesterol and regulated cell-cell adhesion [24,25]. In the present study, we measured changes in cellular cholesterol concentrations by lysophosphatidylcholine in mixed micelles.

In addition to the above (i) and (ii), the following was studied. 

In addition to the anti-rickets activity, in recent years, various biological functions of vitamin D have been attracting attention. A low vitamin D blood level or deficiency reportedly is linked with a heightened risk of various diseases/illnesses, including pregnancy diseases [29,30,31,32], virus diseases [33,34,35,36,37,38], cancers [39,40,41,42,43,44,45,46,47,48], vascular diseases [49,50,51,52], pulmonary diseases [53,54,55,56,57,58], metabolic disorders [59,60,61,62,63], cognition disorders [64,65,66,67,68,69], psychiatric disorders [70,71,72,73], reduction of exercise capacity [74,75], and reproductive function [76,77]. A relationship between maternal plasma levels of vitamin D during pregnancy and development of attention-deficit hyperactivity disorder-like symptoms in offspring has also been suggested [78,79]. It is also believed that the immunomodulatory effects of vitamin D may improve the clinical signs of coronavirus disease 2019 (COVID-19) patients at this very moment [80]. However, vitamin D itself has no biological activity but can exert its function after being metabolically converted; that is, it is activated by introducing an OH group at the C_25_-location in the liver and at the C_1_-location in the kidney. The amounts obtained in organic synthesis of vitamins D_5_–D_7_ used in the present study were very small. To subject these vitamins D to any functional test, the vitamin D needs to be further converted into an activated form, but it was difficult for us to secure adequate amounts. Therefore, we estimated the biological activities of vitamin D by using Prediction of Activity Spectra for Substances (PASS) online software, as we have used in the past [81]. However, there is no intention here to regard activated vitamins D as drugs, but only to discuss the biological activities after ingestion as food components.

## 2. Materials and Methods

### 2.1. Materials

Vitamin D_2_ was purchased from Nacalai Tesque (Kyoto, Japan). Vitamin D_3_ was purchased from Tokyo Chemical Industry Co., Ltd. (Tokyo, Japan). Vitamins D_5_–D_7_ were organically synthesized as described in our previous report [27]. Vitamin D_4_, β-sitosterol, vitamin K_1_, Trolox, fetal bovine serum (FBS), and 1-16:0-2-OH-*sn*-glycerol-3-phosphocholine (lysophosphatidylcholine) were purchased from Sigma-Aldrich (St. Louis, MO, USA). The FBS was heat-inactivated and then used. Dulbecco’s Modified Eagle’s Medium (DMEM) was purchased from Nissui Pharmaceutical (Tokyo, Japan). The other chemicals and solvents were of reagent grade.

### 2.2. Cell Culture and Differentiation

Caco-2 cells were purchased from the American Type Culture Collection (Rockville, MD, USA). DMEM containing 10% FBS, 4 mM L-glutamine, penicillin and streptomycin (40 units/mL and 40 μg/mL, respectively), and nonessential amino acids (0.1 mM) were used as the culture medium. The cells were grown in the culture medium at 37 °C in a humidified atmosphere of 5% CO_2_ in air and then were cultured by passaging two times per week. We used differentiated Caco-2 cells, which have been widely used as a human intestinal model, for vitamin D uptake experiments. To induce differentiation of Caco-2 cells, the cells were seeded in culture plate of 24-well at a density of 5 × 10^4^ cells per well containing 0.5 mL of the culture medium. Then, the medium was changed with fresh medium two times per week for 20–22 d. We have previously reported that the cell monolayers obtained by this method had sufficient cell-cell adhesion [24].

### 2.3. Preparation of Mixed Micelles Containing Vitamin D

The mixed micelles were prepared as described in our previous reports [22,23,24,25]. All of the components, i.e., taurocholate, monoolein, oleic acid, lysophosphatidylcholine, and vitamin D, were dissolved in an appropriate solvent. Portions of these solvent solutions were moved to glass tubes. The solvent was dried up with argon gas and further centrifugal evaporator. The dry matter was dispersed into FBS-free DMEM (same as the culture medium described above but without FBS). To remove any vitamin D not solubilized in the micelles, the resulting solution was passed through a filter (the pore size was 0.2-μm). The materials of mixed micelle and their concentrations were taurocholate (2 mM), monoolein (100 μM), oleic acid (33 μM), lysophosphatidylcholine (0 or 50 μM), and vitamin D (1 μM), respectively, unless otherwise stated. The concentrations were same as those used in our previous study [22,23,24]. Before adding the mixed micelles to the Caco-2cells, the initial concentration of vitamin D was checked as follows. One part of the mixed micelles was diluted with four parts of methylene chloride/methyl alcohol (1:4, *v*/*v*), and then a portion (30 μL) was analyzed by high-pressure liquid chromatography (HPLC) as explained in the “2.8. HPLC Analysis” subsection below. The initial concentration throughout all experiments was 1.04 ± 0.06 μM.

### 2.4. Evaluation of Vitamin D Uptake by Differentiated Caco-2 Cells from Mixed Micelles

Differentiated Caco-2 cell monolayers were washed two times with FBS-free DMEM (0.5 mL) and then treated with medium consisting of mixed micellar vitamin D (0.5 mL) at 37 °C for 2 h in a CO_2_ incubator. After treatment, the following operations were continued to measure i) the amount of vitamin D uptake by the cells and ii) the residual amounts of mixed micellar vitamin D in the medium.

(i): The cells were washed with 0.5 mL of 10 mM taurocholate in phosphate-buffered saline (PBS) and then washed two times with PBS. They were homogenized in 1 mL PBS containing 0.01 mL of 0.2 mM Trolox/methyl alcohol, which is an antioxidant, by using a probe-type sonicator. An aliquot (0.8 mL) of the cell homogenate was mixed with 0.2 mL of 1 μM vitamin K_1_/ethyl alcohol, which was used as an internal standard. Subsequently, ethyl alcohol (0.6 mL), ethyl acetate (0.8 mL), and *n*-hexane (0.8 mL) were added, and the mixture was shaken with a Vortex mixer after each addition. The upper layer was collected, and ethyl acetate and *n*-hexane were added to the lower layer and shaken in the same manner. This upper layer was combined with the first upper layer. The combined upper layers were dried in a centrifugal evaporator. The extract was dissolved in 200 μL of methylene chloride/methyl alcohol/H_2_O (9:81:10, *v*/*v*/*v*), and an aliquot (40 μL) was then subjected to HPLC as described in the “2.8. HPLC Analysis” subsection below. The recovery of the vitamin D spiked into a suspension of differentiated Caco-2 cells was 100.3% ± 1.2% (*n* = 4). To determine the cellular protein content, an aliquot (50 μL) of the cell homogenate was subjected to the Lowry method [82].

(ii): The micellar vitamin D decreased by agglomeration might have influenced the uptake of vitamin D from mixed micelles to cells because only micellar vitamin D could be taken up by Caco-2 cells, as shown by the agglomeration of carotenoids described in our previous report [22]. Parts of the medium collected at the end of the incubation were passed through a filter, as described above. One part filtrate was diluted with four parts methylene chloride/methyl alcohol (1:4, *v*/*v*), and then 40-μL was injected for HPLC analysis as described in the “2.8. HPLC Analysis” subsection below.

### 2.5. Evaluation of Facilitated Diffusion on Vitamin D Uptake by Differentiated Caco-2 Cells from Mixed Micelles

Although involvement of facilitated diffusion in intestinal absorption of vitamin D3 has been previously reported [83], other vitamins D have not been tested. In the present study, we evaluated the involvement of facilitated diffusion in the cellular uptake of each mixed micellar vitamin D_2_–D_7_ by using ezetimibe, an inhibitor of the protein Niemann-Pick C1-like 1 (NPC1L1) [84]. Ezetimibe was dissolved in dimethyl sulfoxide (DMSO), and then was added at a final concentration of 150 μM to the culture medium. At this time, the final concentration of DMSO was 1.5% (*v*/*v*). The differentiated Caco-2 cells were preincubated at 37 °C for 2 h with ezetimibe-containing culture medium (0.5 mL). The cells were washed two times with FBS-free DMEM (0.5 mL) and then treated with the medium containing mixed micellar vitamin D (0.5 mL) for 2 h, as described above. After treatment, cellular vitamin D, residual mixed micellar vitamin D, and the cellular protein content were evaluated as described in the previous subsection. 

### 2.6. Impact of Cell-Cell Adhesion/Cell-Matrix Adhesion in Caco-2 Cells on Vitamin D Uptake

Since 2001, we have reported that lysophospholipids enhance the uptake of micellar carotenoids in Caco-2 cells [22,23,24,85]. In 2015, we demonstrated that cell-cell adhesion and cell-matrix adhesion were involved in the mechanism of its enhancing effect [24]. In the present study, using the same method, we examined whether the mechanism for the enhancing effect of lysophosphatidylcholine could be adapted to vitamin D, as well. To evaluate the impact of the degree of cell-cell adhesion/cell-matrix adhesion on vitamin D uptake by cells, we prepared in two different ways: i) dispersed Caco-2 cells without cell-cell adhesion/cell-matrix adhesion and ii) Caco-2 cells with insufficient cell-cell adhesion.

(i): The dispersed cells were prepared by trypsin treatment. After collecting the cells in the culture medium, the number of cells was counted and diluted with the medium. The cells were washed two times with FBS-free DMEM and then seeded in culture plate of 24-well for suspension cultures (SC plate; Greiner Bio-One, Frickenhausen, Germany) at a density of 15 × 10^4^ cells per well containing the FBS-free DMEM (0.5 mL).

(ii): The cells that had insufficient cell-cell adhesion were prepared by reducing the culture period from 20–22 d for full differentiation to <7 d after the seeding described above.

Subsequent operations are common to the two models of cells. The medium consisting of mixed micellar vitamin D (0.5 mL) was added to adhered cells or dispersed cells in individual wells containing FBS-free DMEM (0.5 mL), resulting in a total medium volume of 1.0 mL/well, and the final start concentrations of mixed micellar components were one-half the initial concentration. The cells were incubated in the same conditions as described above, i.e., in a CO_2_ incubator at 37 °C for 2 h, and then the cells were washed with 2 mM taurocholate/PBS, followed by two times with PBS. The vitamin D uptake and residual mixed micellar vitamin D were evaluated as the previous subsection, but the filtrate of residual mixed micellar vitamin D was diluted with a 2x-volume of H_2_O. The cellular protein content was also determined as above.

### 2.7. Evaluation of Cellular Cholesterol in the Differentiated Caco-2 Cells Treated with the Mixed Micelles Containing Lysophosphatidylcholine

Mixed micelles were prepared and treated with the cells in the same manner as in the experiment using differentiated Caco-2 cells, but the concentration of lysophosphatidylcholine was varied from 0 to 250 μM, and the micelles did not include vitamin D. After treatment, the cells were washed and homogenized. The saponification reaction was performed as follows. A portion (0.8 mL) of the cell homogenate was mixed with 200 μL of 40 μM β-sitosterol/ethyl alcohol as an internal standard, 85 μL of 10 M KOH, and 0.6 mL ethyl alcohol and then incubated at 60 °C for 2 h under argon gas. After cooling the reaction solution, cholesterol was extracted with ethyl acetate and *n*-hexane in the same manner as the extraction of vitamin D described above. The extract was additionally washed with 0.8 mL H_2_O before the solvent completely dried and then dissolved in 200 μL of methylene chloride/methyl alcohol/H_2_O (9:81:10, *v*/*v*/*v*). An aliquot (40 μL) was then subjected to HPLC as described in the “2.8. HPLC Analysis” subsection below. The cellular protein content was also determined as above. 

### 2.8. HPLC Analysis

Vitamin D and cholesterol were analyzed by using the HPLC system and columns which were the same as in our previous report [24]. Mobile phase X was ethanenitrile/methyl alcohol/H_2_O (75:15:10, *v*/*v*/*v*), mobile phase Y was methyl alcohol/ethyl acetate (70:30, *v*/*v*), and mobile phase Z was methyl alcohol/isopropyl alcohol (70:30, *v*/*v*). These mobile phases contained 0.1% ammonium acetate. An isocratic analysis was performed at a flow rate of 0.2 mL/min with mobile phase X/Y (30:70, *v*/*v*) for vitamin D or mobile phase X/Z (30:70, *v*/*v*) for cholesterol. Vitamin D and cholesterol were quantified from the peak area at 265 nm and 210 nm, respectively, by using the respective calibration curve of the authentic standard.

To minimize degradation of vitamin D by light irradiation, all experiments were carried out under dim yellow light.

### 2.9. Statistical Methods

The data were analyzed by one-way analysis of variance, followed by the *t*-test, Dunnett test, or Tukey–Kramer test. *p*-values < 0.05 were considered significant.

### 2.10. Estimation of Biological Activity of Vitamin D by Online Software Simulation

According to the homepage (http://www.pharmaexpert.ru/passonline/, accessed on 26 March 2021), the biological activity of organic compounds can be predicted by PASS online software. Vitamin D is metabolized into 1,25-di(OH)-vitamin D of an activated form through a two-step metabolism. Thus, in this study, the biological activity of activated form was predicted by PASS online software, as we have previously examined with another component [81]. The result was output in both Pa values (probability to be active) and Pi values (probability to be inactive). The closer the Pa value is to 1, the higher the activity. The larger the Pi value, the more inactive it is. The biological activities shown by the Pa and Pi values among vitamins D_2_–D_7_ were compared.

## 3. Results

Mixed micelles without and with lysophosphatidylcholine were designated NoPC mixed micelles and lysoPC mixed micelles, respectively. In our previous studies on the carotenoid absorption by Caco-2 cells, the concentration of residual micellar carotenoids after incubation was lower than the theoretical value, especially in NoPC mixed micelles [22,23,24]. One concern was that the reduction of micellar carotenoids might reduce the carotenoid transfer from micelles into cells; in other words, this decrease might be a factor affecting cellular uptake. However, we previously investigated this issue and dispelled the concern [24,25]. In the present study, as well, the decrease in micellar vitamin D during incubation did not seem to affect the cellular uptake, but, as a precaution, the residual micellar vitamin D concentration was monitored.

### 3.1. Vitamin D Uptake by Differentiated Caco-2 Cells and Effect of Lysophosphatidylcholine on Uptake

**Figure 2:**
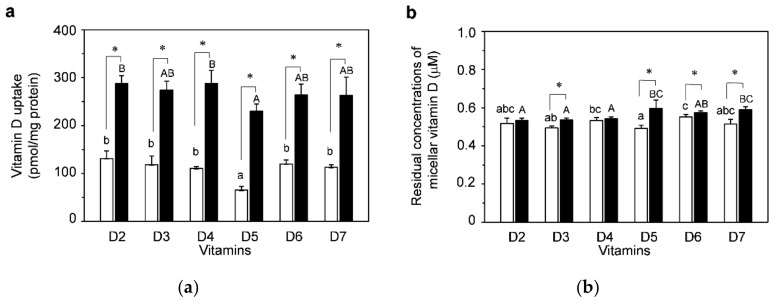
Uptake of vitamin D solubilized in mixed micelles by differentiated Caco-2 cells and the effects of lysophosphatidylcholine. Differentiated Caco-2 cells were incubated for 2 h with vitamin D in mixed micelles without lysophosphatidylcholine and mixed micelles with lysophosphatidylcholine, called NoPC mixed micelles (open bars) and lysoPC mixed micelles (filled bars), respectively. (**a**) Vitamin D uptake by differentiated Caco-2 cells. (**b**) Residual concentrations of vitamin D in the mixed micelles (micellar vitamin D) after 2-h incubation with the cells. Data are presented as the means ± standard deviation of four wells in a single experiment. Replicate experiments showed a similar trend. Statistical analyses were performed between NoPC mixed micelle and lysoPC mixed micelle in same vitamin D, among 6 kinds of vitamins D in NoPC mixed micelles, and among 6 kinds of vitamins D in lysoPC mixed micelles in each graph. The asterisk indicates a value with significant difference as determined by unpaired *t*-test (*p* < 0.05). Values not sharing a common letter were significantly different as determined by Tukey–Kramer test (*p* < 0.05).

Figure 2a shows a comparison of uptakes of vitamins D_2_–D_7_ solubilized in NoPC mixed micelles (open bars) and lysoPC mixed micelles (filled bars) by differentiated Caco-2 cells. The uptake amount was lower by vitamin D_5_ than by other vitamins D in the NoPC mixed micelles. Compared with NoPC, lysophosphatidylcholine markedly enhanced all of the vitamin D uptakes (2.5 times on average for six vitamin D species). The vitamin D uptakes in lysoPC mixed micelles showed a trend similar to that in NoPC mixed micelles; that is, vitamin-D_5_ uptake tended to be lower than that of other vitamins D, but not statistically significantly different from that of vitamins D_3_, D_6_, and D_7_.

Figure 2b shows the residual concentrations of micellar vitamin D in the medium after treatment of the Caco-2 cells with vitamin D. More than half of their initial concentrations remained in all vitamins D micelles. In vitamins D_3_, D_5_, and D_7_ micelles, the amount was statistically higher for lysoPC than for NoPC.

### 3.2. Possible Involvement of Facilitated Diffusion in Vitamin D Uptake

**Figure 3:**
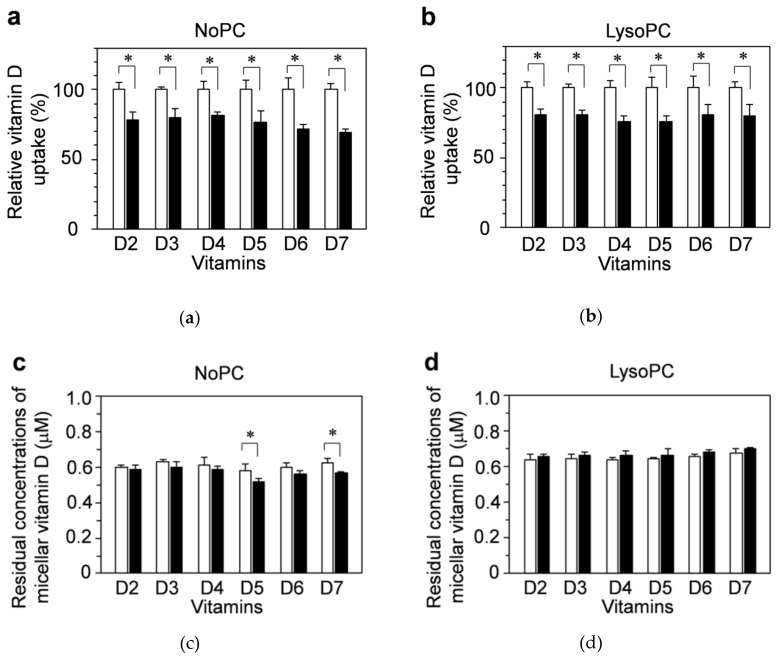
Inhibitory effects of ezetimibe on the uptake of vitamin D solubilized in mixed micelles by differentiated Caco-2 cells. Differentiated Caco-2 cells were pretreated for 2 h with the vehicle (1.5% dimethyl sulfoxide (DMSO), open bars) alone or with 150 μM ezetimibe (filled bars), which is an inhibitor of the protein Niemann-Pick C1-like 1 (NPC1L1), and then incubated for 2 h with vitamin D in NoPC mixed micelles (left side) and lysoPC mixed micelles (right side). (**a**,**b**): Vitamin D uptake by differentiated Caco-2 cells. (**c**,**d**): Residual concentrations of micellar vitamin D after 2-h incubation with the cells. Data are presented as the means ± standard deviation of four wells in a single experiment. Replicate experiments showed a similar trend. Statistical analysis was performed to compare each vitamin D with and without ezetimibe. The asterisk indicates a value with significant difference as determined by unpaired *t*-test (*p* < 0.05).

We evaluated the involvement of facilitated diffusion in the cellular uptake of vitamins D_2_–D_7_ by using an inhibitor (ezetimibe) that blocked part of the diffusion. Differentiated Caco-2 cells were pretreated with culture medium containing vehicle (1.5% DMSO) alone as a control (open bar) or 150 μM ezetimibe (filled bar) for 2 h, and then NoPC mixed micelles (a,c) or lysoPC mixed micelles (b,d) containing vitamin D were added to the cells. The vitamin D uptake amount was calculated relative to the control. Ezetimibe inhibited the uptakes of vitamins D_2_–D_7_ by approximately 20%. Similar trends were shown for the NoPC mixed micelles (Figure 3a) and lysoPC mixed micelles (Figure 3b).

There were no differences in the concentrations of residual micellar vitamin D except for vitamins D_5_ and D_7_ in the NoPC mixed micelles (Figure 3c,d).

### 3.3. Impact of Cell-Cell Adhesion/Cell-Matrix Adhesion on Vitamin D Uptake by Caco-2 Cells

We previously reported on the mechanism by lysoglycerolipids, enhanced the cellular uptake of carotenoids [24]. Briefly, the mechanism underlying the enhancing effect involved cell-cell adhesion/cell-matrix adhesion rather than cell membrane permeability. In the present study, we investigated the impact of cell-cell adhesion/cell-matrix adhesion on vitamin D uptake by using a similar approach.

As shown in Figure 2a, differences were observed in the uptake amounts among the six types of vitamin D in both NoPC mixed micelles and lysoPC mixed micelles. The effect of cell-cell adhesion/cell-matrix adhesion that contributed to the difference in uptake among these vitamins D was evaluated in dispersed Caco-2 cells, which were prepared by trypsin treatment. Because the dispersed cells had no cell-cell adhesion/cell-matrix adhesion, we could assess its effect by comparison with the results obtained with differentiated Caco-2 cells.

**Figure 4:**
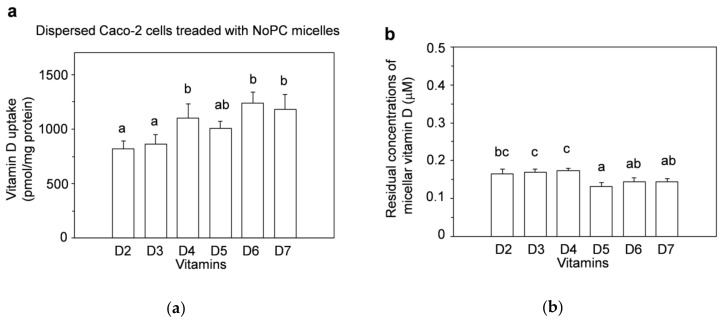
Uptake of vitamin D solubilized in mixed micelles by dispersed Caco-2 cells. Dispersed Caco-2 cells without cell-cell adhesion/cell-matrix adhesion were incubated for 2 h with vitamin D in NoPC mixed micelles. The cells were prepared by dispersing the cells with trypsin. (**a**) Vitamin D uptake by dispersed Caco-2 cells. (**b**) Residual concentrations of the micellar vitamin D after 2-h incubation with the cells. Data are presented as the means ± standard deviation of four wells in a single experiment. Replicate experiments showed a similar trend. Statistical analysis was performed among all six samples in each graph. Values not sharing a common letter were significantly different as determined by Tukey–Kramer test (*p* < 0.05).

The uptake by dispersed Caco-2 cells (Figure 4a) was different from that of differentiated Caco-2 cells that were cultured for 20–22 d (Figure 2a), there was no tendency toward decreased vitamin-D_5_ uptake, and the uptake amounts of vitamins D_4_, D_6_, and D_7_ were higher than those of vitamins D_2_ and D_3_ (Figure 4a).

As described in the Materials and Methods section, the initial concentration of vitamin D in the mixed micelles was now half that of those in the differentiated Caco-2 cells because of the 2-fold dilution. However, the absolute amounts contained in the medium were the same in both experiments. The residual concentrations of micellar vitamin D in the medium tended to be lower in vitamins D_5_–D_7_, but approximately ≥0.3 μM remained in all types of mixed micelles (Figure 4b).

### 3.4. Cell-Cell Adhesion/Cell-Matrix Adhesion for Enhancing Effects of Micellar Lysophosphatidylcholine on Vitamin D Uptake: Examination in Dispersed Cells

Then we investigated the participation of cell-cell adhesion/cell-matrix adhesion in the enhancing effects of lysophosphatidylcholine on vitamin D uptake by using the dispersing Caco-2 cells. In the following experiments, vitamin D_2_ was used as a representative.

**Figure 5:**
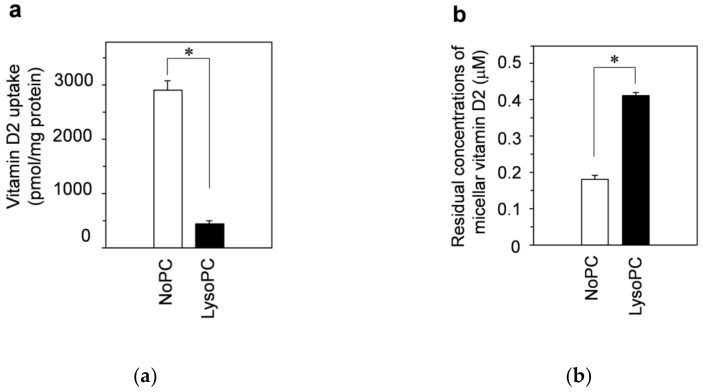
Effects of lysophosphatidylcholine on the uptake of vitamin D_2_ solubilized in mixed micelles by dispersed Caco-2 cells. Dispersed Caco-2 cells without cell-cell adhesion/cell-matrix adhesion were incubated for 2 h with vitamin D_2_ in NoPC mixed micelles and lysoPC mixed micelles. (**a**) Vitamin-D_2_ uptake by dispersed Caco-2 cells. (**b**) Residual concentrations of micellar vitamin D_2_ after 2-h incubation with the cells. Data are presented as the means ± standard deviation of four wells in a single experiment. Replicate experiments showed a similar trend. Statistical analysis was performed among all six samples in each graph. The asterisks indicate significant differences as determined by unpaired *t*-test (*p* < 0.05).

The open bars and filled bars in Figure 5a show the vitamin-D_2_ uptake amounts from the NoPC mixed micelles and lysoPC mixed micelles, respectively. The effect of lysophosphatidylcholine on the vitamin-D_2_ uptake by dispersed Caco-2 cells was much different from that of the differentiated Caco-2 cells. No enhancing effect of lysophosphatidylcholine on vitamin-D_2_ uptake by dispersed cells was observed. The vitamin-D_2_ uptake from lysoPC mixed micelles was approximately one-sixth that from NoPC mixed micelles.

The residual concentrations of micellar vitamin D_2_ in the medium were lower in NoPC mixed micelles than in lysoPC mixed micelles (Figure 5b).

### 3.5. Cell-Cell Adhesion/Cell-Matrix Adhesion for Enhancing Effects of Lysophosphatidylcholine on Vitamin D Uptake: Examination Using Adherent Caco-2 Cells with Insufficient Cell-Cell Adhesion

In addition to evaluations in dispersed cells, we evaluated the uptake of vitamin D_2_ in cells with insufficient cell-cell adhesion. The cells were cultured for 3, 5, and 7 d after seeding into 24-well plates and then incubated with vitamin D_2_ solubilized in mixed micelles.

**Figure 6:**
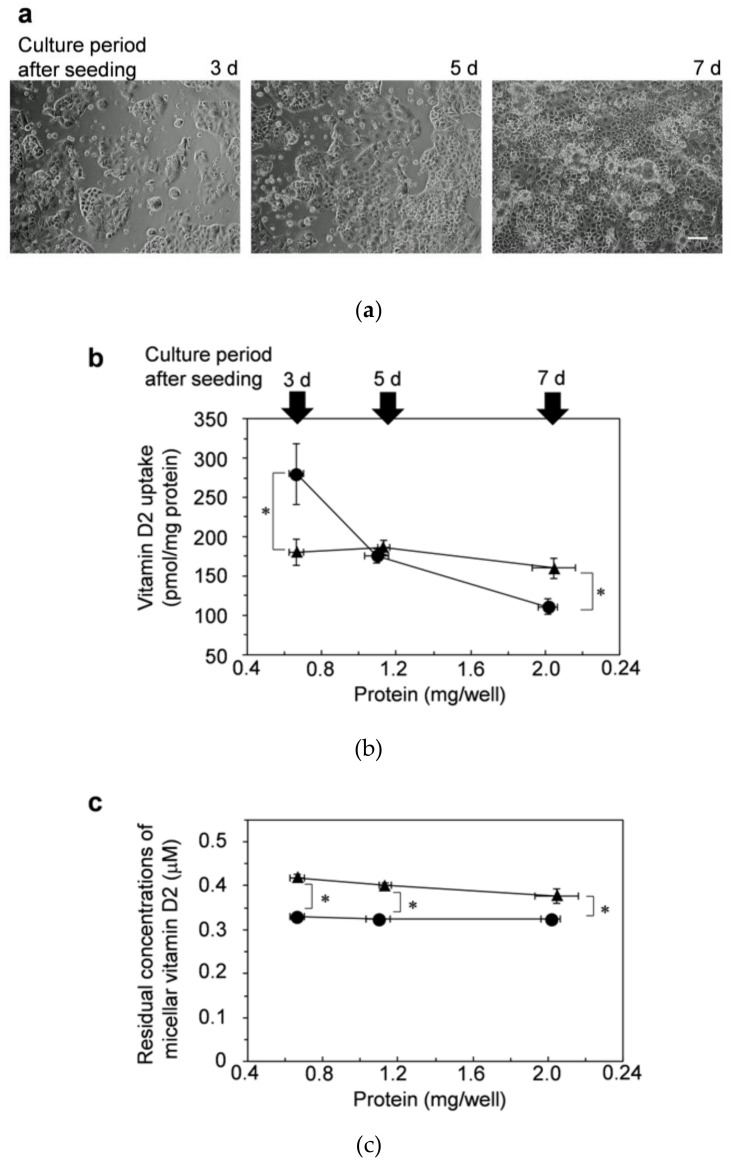
Effects of lysophosphatidylcholine on the uptake of vitamin D_2_ by Caco-2 cells with insufficient cell-cell adhesion. Caco-2 cells with insufficient cell-cell adhesion/cell-matrix adhesion were prepared by cultivating for 3, 5, and 7 d after seeding in the culture plate. (**a**) These cells were observed by phase-contrast microscopy (100×). The white line in the photo of 7 d indicates the scale bar (100 μm). The adhered Caco-2 cells were then incubated for 2 h with vitamin D_2_ in NoPC mixed micelles (circles) and lysoPC mixed micelles (triangles). (**b**) Vitamin-D_2_ uptake by the adherent Caco-2 cells. (**c**) Residual concentrations of the micellar vitamin D_2_ after 2-h incubation with the cells. Data are presented as the means ± standard deviation of four wells in a single experiment. Replicate experiments showed a similar trend. Statistical analysis was performed between two mixed micelles at the same cultivation time (3, 5, and 7 d). The asterisk indicates a value with significant difference as determined by unpaired *t*-test (*p* < 0.05).

As shown in Figure 6a, the cells adhered to the bottom of a plate, but there is a distance between the cell colonies cultured for 3 d. The distance was shortened after 5 d of culture. Finally, the cells reached confluence after cultivating for 7 d. Then, the cells cultured for 3 d and 5 d had insufficient cell-cell adhesion.

The results shown by using cells cultured for 3 d (Figure 6b) were also much different from those shown by using the differentiated cells, as shown in Figure 2a. The amount of vitamin D uptake was significantly higher by cells cultured for 3 d from NoPC mixed micelles (circle) than those from lysoPC mixed micelles (triangle). However, the uptake from NoPC mixed micelles decreased as the culture time increased to 5 d and 7 d. In contrast, the amounts of vitamin D_2_ uptake from lysoPC mixed micelles showed little change (Figure 6b). There was no difference in the uptake amounts between NoPC mixed micelles and lysoPC mixed micelles in cells cultured for 5 d. Finally, in the cells cultured for 7 d, the amounts of vitamin D_2_ uptake from lysoPC mixed micelles were significantly higher than those from NoPC mixed micelles, similar to the tendency of the results by the differentiated Caco-2, as shown in Figure 2a.

The residual concentrations of micellar vitamin D_2_ in the medium was lower in NoPC mixed micelles (circle) than in lysoPC mixed micelles (triangle) in cell-culture periods from 3–7 d (Figure 6c).

### 3.6. Effect of Lysophosphatidylcholine in Mixed Micelles on Cellular Cholesterol Amounts in Differentiated Caco-2 Cells

**Figure 7:**
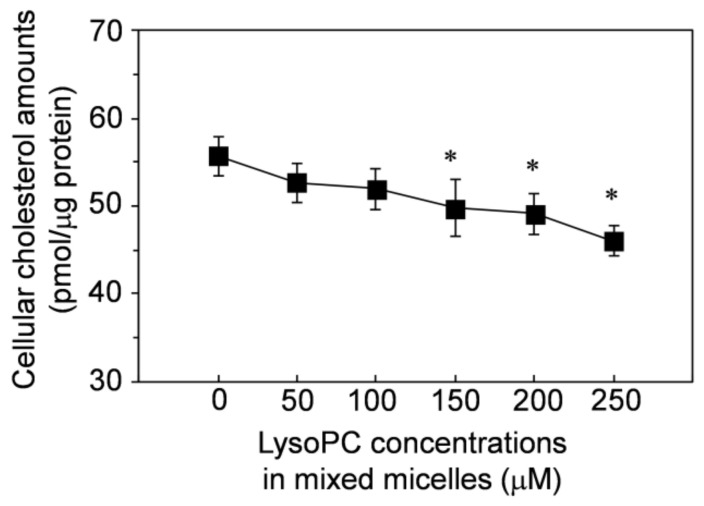
Reducing effect of lysophosphatidylcholine in mixed micelles on cellular cholesterol amounts in the differentiated Caco-2 cells. The differentiated Caco-2 cells were incubated for 2 h with the mixed micelles containing lysophosphatidylcholine at 0–250 μM. The mixed micelles contained no vitamin D. Data are presented as the means ± standard deviation of four wells in a single experiment. Replicate experiments showed a similar trend. The asterisks indicate significant differences from the value of lysophosphatidylcholine at 0 μM as determined by Dunnett’s test (*p* < 0.05).

Lysophosphatidylcholine may regulate the amount of cholesterol originally contained in cells (cellular cholesterol), thereby affecting cell-cell adhesions and enhancing vitamin D uptake. We evaluated whether lysophosphatidylcholine in mixed micelles affected the amounts of cellular cholesterol. As shown in Figure 7, the amounts of cellular cholesterol decreased depending on the concentration of lysophosphatidylcholine in the mixed micelles. Lysophosphatidylcholine at >150 μM significantly decreased the cellular cholesterol and even concentrations <100 μM still tended to decrease the cellular cholesterol, but the decrease was not significant.

### 3.7. Estimation of Biological Activities of 1,25-Di(OH)-Vitamin D

Ingested vitamin D is absorbed from the intestinal tract and a portion is metabolized to 1,25-di(OH)-vitamin D by hydroxylation of the C_25_-location in the liver and further hydroxylation of the C_1_-location in the kidney. We analyzed the biological activities of the respective activated forms of vitamins D_2_–D_7_ by PASS online software simulation.

The PASS simulation showed the diverse biological activities that vitamins D possess, as described in the introduction section. However, only the biological activities associated with vitamin D as an anti-rickets factors are listed here in Table 1. Since PASS cannot distinguish between vitamins D_4_ and D_7_ (these chemical structures are shown in Figure 1), the same results were output. Among the five types of vitamin D (D_2_, D_3_, D_4_/D_7_, D_5_, and D_6_), the one with the highest Pa value was marked with “a,” and the runner-up was “b.” For each vitamin D, Pa was output at a higher value for bone-/calcium regulation-related activities (Table 1). In “vitamin” and “bone-formation stimulant”, the Pa value was higher for vitamin D_5_ than for vitamins D_2_ and D_3_. Vitamin D_6_ had the highest Pa value after vitamin D_2_ in “calcium regulator”, “vitamin D-like”, and “vitamin D receptor agonist”. 

## 4. Discussion

To investigate the possibility of increasing the chance of vitamin D ingestion, we assumed the intestinal absorption of vitamins D_4_–D_7_ in addition to vitamins D_2_ and D_3_ and compared the uptake characteristics of these vitamins D solubilized in mixed micelles by using an intestinal cell model with differentiated Caco-2 cells. Except for vitamin D_5_, these vitamins D showed no significant differences in the amounts taken up by the cells (Figure 2). This result suggested that after vitamin D was solubilized in mixed bile salt micelles in the intestinal tract, vitamins D_4_, D_6_, and D_7_ were taken up by intestinal cells at levels similar to those for vitamins D_2_ and D_3_. In a previous study, we reported that the tendency of cellular uptake by Caco-2 for 11 kinds of carotenoids was close to their actual human bioavailability [85]. For example, α-carotene, β-carotene, lutein, β-cryptoxanthin, and zeaxanthin had a high uptake rate by Caco-2 cells, and in fact, these are typical carotenoids present in human blood [86]. On the other hand, neoxanthin and fucoxanthin had very low cellular uptake and are hardly absorbed by humans [86]. The results for vitamin D_4_–D_7_ uptake cannot be compared with their human bioavailability, since there are no human test results. This may be worth considering in future studies. In addition to the uptake, the study of vitamins D secretion using Transwell could also be an issue for future study.

As much as uptake, solubilization of vitamin D into mixed bile salt micelles is also an essential factor in its bioavailability. However, solubilization of vitamins D_4_–D_7_ in food is an issue for future research because the foods containing vitamins D_4_–D_7_ are not well known even now. Vitamin D_2_ is present in mushrooms. Vitamin D_3_ is mainly found in fish and shellfish; shiitake mushrooms [87]; and leaves of plants, such as tomatoes, eggplants, and zucchini [88,89]. Unfortunately, vitamin D_3_ was not found in these edible fruit parts. As mentioned above, in 1924, alfalfa was reported to contain an anti-rickets factor [11]. In 1984, the factors were identified as vitamins D_2_ and D_3_ [90]. However, plants cannot synthesize ergosterol. Vitamin D_2_ in plants has been identified as a contaminant derived from fungi (mold) [91]. Therefore, vitamin D_3_ is probably the main form in alfalfa. Although vitamin D_4_ was isolated in 1937 [92] at the same time as the isolation of vitamin D_3_ [93], it was not until 2012 that the foods containing it were revealed. Vitamin D_4_ is also contained in mushrooms [94]. Vitamin D_5_ and its precursor (7-dehydrositosterol) were reported to be present in *Arabidopsis thaliana* in 2018, and UV irradiation has been shown to increase its amount of vitamin D_5_ [95]. In addition, 7-dehydrositosterol in natural products is known to be present in *Cyanidium caldarium* (red algae) [96] and *Rauwolfia serpentina* [97]. These would have the potential to contain vitamin D_5_. There are few reports on what foods contain vitamins D_6_ and D_7_, but there are reports about the presence of their precursors in nature. Since 7-dehydrostigmasterol (vitamin D_6_ precursor) or 7-dehydrocampesterol (vitamin D_7_ precursor) is present in protists, such as trypanosomes [98] and amoeba [99] or in *Critidia* (in flagella) [98], vitamins D_6_ and D_7_ must be produced by UV irradiation in nature. Both stigmasterol and campesterol are typical plant sterols. In the future, their 7-dehydro forms, or vitamins D_6_ and D_7_, may be found in some foods, which would increase vitamin D intake opportunities.

Ergosterols and plant sterols are not absorbed in the intestine [100,101]. Like ergosterol, the 7-dehydro form of plant sterols will not be absorbed in the intestinal tract. If they are absorbed, they must be transported to the skin and exposed to UV irradiation to be converted to vitamin D forms. To the best of our knowledge, no such route has been reported. Therefore, we have to ingest the vitamin D form, not the precursor.

Intestinal absorption and accumulation of vitamin D seems to vary depending on the species. Both vitamins D_2_ and D_3_ are absorbed in humans, but the absorbability has not been determined because that of D_2_ equals that of D_3_ [87,102,103] or D_3_ > D_2_ [104,105] has been reported. Although fish ingest both vitamins D_2_ and D_3_ from plankton, most vitamin D_3_ is found in the body of the fish [106,107], and vitamin D_2_ is hard to accumulate. In chicks, vitamin D_2_ is less absorbed than vitamin D_3_ [108] or D_4_ [109]. In rats, vitamin D_2_ is slightly more easily absorbed than vitamin D_3_ [108], and vitamins D_3_ and D_4_ have similar levels of absorption [109]. Little information is available on the intestinal absorbability of vitamins D_5_–D_7_. The present study may be one of the hints of the little information.

The uptake of vitamin D_5_ tended to be smaller than that of other vitamins D. In addition to the uptake by differentiated Caco-2 cells, we evaluated the uptake by dispersed Caco-2 (Figure 4). The test with dispersed cells was originally intended to investigate the impact of cell-cell adhesions on cellular uptake, but simultaneously, we considered that this reflected the ease with which vitamin D could be transferred from mixed micelles to cell membrane lipids, as reported previously [24]. However, the uptake by dispersed cells did not match that by differentiated cells, and the dispersed cell experiment did not show that vitamin D_5_ was less likely than other vitamins D to be absorbed. The basic skeleton on the chemical structure of vitamin D is secosterol, and the types of vitamin D are determined by differences in their side chain structure (Figure 1). The side chains of vitamin D_5_ and β-sitosterol, a plant sterol, are the same. In the uptake test of plant sterols by Caco-2 cells, the order of increasing uptake was reported to be campesterol > β-sitosterol = brassicasterol ≥ stigmasterol [110]. The absorption characteristics may differ between vitamins D and plant sterols even with the same side chains. Thus, the trend of the vitamin D amounts taken up by differentiated cells may be caused by factors other than the transfer of vitamin D from mixed micelles to cell membrane lipids.

Niemann-Pick Cl-like 1 (NCP1L1) and scavenger receptor class B member 1 (SR-B1) are a transporter and a receptor, respectively, related to the intestinal absorption of cholesterol [111]. Reportedly, these cholesterol absorption-related factors are also involved in absorption of vitamin D_3_, which has the same side chain as cholesterol, in Caco-2 cells and mice [83]. Ezetimibe is widely used as an inhibitor of NCP1L1 [84]. We used ezetimibe to investigate the involvement of the facilitated diffusion pathway via NCP1L1 in vitamin D uptake. We found that the vitamin-D_3_ uptake was blocked by ezetimibe at an approximate rate of 20–30%, a finding similar to a previous one [83]. The uptakes of vitamins D_2_ and D_4_–D_7_ showed tendencies similar to that of vitamin D_3_ (Figure 3), suggesting that facilitated diffusion via the NCP1L1 pathway occurred in 20–30% of the uptakes of the vitamin D forms tested. Although the NCP1L1 pathway was not intervened in the low vitamin-D_5_ uptake by differentiated Caco-2 cells, the involvement of SR-B1 was not examined here but will be studied in the future.

Another possibility for low vitamin-D_5_ uptake by the differentiated Caco-2 cells is that an excretory transporter may be involved. Plant sterols are well known substrates for excretory transporters and ATP-binding cassette transporters G5/G8 (ABC G5/G8) [101]. Recently, vitamin-D_3_ absorption was reported to increase in mice deficient in ABC G5/G8 relative to that of the wild type [112], indicating that some vitamin D_3_ absorbed in the intestinal tract is excreted luminally by ABC G5/G8 in mice. Since the involvement of excretory transporters in other types of vitamin D is unknown, this point will also be studied in the future.

As mentioned above, foods containing vitamin D are limited. However, the deficiency of vitamin D intake due to low-intake opportunities might be recoverable by increasing the intestinal absorbability of vitamin D through the absorption-enhancing effect. We have shown in previous studies that lysoglycerolipids enhanced the intestinal absorption of carotenoids [22,23,24,25]. The applicability of the enhancing effect to vitamin D was examined in a present study. We demonstrated that lysophosphatidylcholine conspicuously enhanced the uptake of vitamin D by differentiated Caco-2 cells, suggesting that the enhancing effect is probably applicable to various fat-soluble components, including carotenoids and vitamins D. In a real diet, the use of phospholipids, such as phosphatidylcholine from egg yolk and soybeans, may increase the bioavailability of vitamin D because phospholipids derived from ingested food were hydrolyzed by pancreatic lipase to free fatty acids and lysophospholipids, such as lysophosphatidylcholine, in the human intestinal tract.

We think that lysophosphatidylcholine caused enhanced vitamin D uptake through the same mechanism underlying carotenoids uptake. We previously reported that the formation of cell-cell adhesion/cell-matrix adhesion is involved in the mechanism [24,25]. In the present study, we evaluated the enhancing effects of lysophosphatidylcholine on vitamin D uptake by cultivating two models of cells with adhesion conditions different from those of the differentiated cells: dispersed Caco-2 cells without cell-cell adhesion/cell-matrix adhesion, and adhered Caco-2 cells with insufficient cell-cell adhesion caused by low-density adherence to the culture plate.

In experiments with dispersed cells, the vitamin D uptake amount was markedly lower from mixed micelles with lysophosphatidylcholine than from those without lysophosphatidylcholine (Figure 5). This result showed two things: the transfer of vitamin D to cell membrane lipids was considerably more efficient from NoPC mixed micelles than from lysoPC mixed micelles, and cell-cell adhesion/cell-matrix adhesion was involved in the enhancing effect of lysophosphatidylcholine. Subsequently, we evaluated the vitamin D uptake by adhered cells with insufficient cell-cell adhesion. The uptake amounts from the NoPC mixed micelles were remarkably reduced depending on the growth of cell-cell adhesion, while those from the lysoPC mixed micelles were not changed. Consequently, when the cell-cell adhesion was fully developed, the trend of both uptake results was reversed, indicating that the growth of cell-cell adhesion reduced uptake. Together with our previous results [24], the enhancing effect of lysophosphatidylcholine would be applied not only to carotenoids but also to vitamins D or other fat-soluble components.

Lysophosphatidylcholine has been previously reported to reduce the transelectrical epithelial resistance (TEER) of Caco-2 cells; that is, to reduce the intercellular barrier formed by cell-cell adhesion [113,114,115,116]. As observed for lysophosphatidylcholine, methyl β-cyclodextrin [117,118,119], dodecylphosphocholine [120], and 2-alkoxy-3-alkylamidopropylphosphocholines [121] also reduced the TEER of Caco-2 cells, and they also enhanced the absorption of hydrophilic components, such as mannitol [121] and dextran [118,121]. At the same time, cellular cholesterol was removed from the cells [117,118,119], which would be involved in the enhancing effect on absorption. Removal of cellular cholesterol by lysophosphatidylcholine enhanced the expression of 3-hydroxy-3-methylglutaryl-coenzyme A reductase and maintained cholesterol levels in the cells [122]. Cellular cholesterol contributed to the formation of the intercellular barrier formed by cell-cell adhesion [119]. The intestinal absorption of hydrophilic components was transported via paracellular diffusion [118,121], which is the intercellular barrier-to-basolateral side transport process.

On the other hand, intestinal absorption of hydrophobic compounds, such as carotenoids and vitamins D, has been shown to be transported via transcellular diffusion across the cell membrane [123]. In our previous study, the enhancing effect of lysophosphatidylcholine did not involve the TEER of Caco-2 cells on intestinal absorption of carotenoid [25]. These studies with transwells can examine secretion in addition to uptake, but conversely, they are not suitable for experiments with cells with immature cell-cell adhesion as we have shown here.

The present study also suggested that lysophosphatidylcholine enhanced the uptake of vitamins D by decreasing the intercellular barrier formation by removal of cholesterol from the cells in the transcellular pathway. We previously showed a figure of the candidate mechanism for the enhancing effect of lysophosphatidylcholine on uptake of carotenoids by Caco-2 cells [24], but we have not shown it again here. This mechanism can extend its application to other fat-soluble components, at least vitamins D, which would have been removable from mixed micelles across the region of the cytoplasmic membrane in dispersed Caco-2 cells. The components would be more easily transferred from NoPC mixed micelles than from lysoPC mixed micelles. In the case of adherent cells, cell-cell adhesion/cell-matrix adhesion would extremely decrease the region through which the components in NoPC mixed micelles could be moved to the cytoplasmic membrane. On the other hand, lysophosphatidylcholine would not decrease the moveable region of the components, which may have been caused by reduction of the formation of cell-cell adhesion/cell-matrix adhesion of Caco-2 cells due to loss of cellular cholesterol by lysophosphatidylcholine. Thus, the uptake level increased more by lysoPC mixed micelles than by NoPC mixed micelles in link with the adhesion growth.

In our previous study, to investigate the involvement of cell membrane permeability as a candidate mechanism for the enhancing effect of lysoglycerolipids, we examined the permeation-promoting effect of lysoglycerolipids by using 5(6)-carboxyfluorescein-encapsulated Phosphatidylcholine liposomes [24]. However, the results suggested that an alteration in the membrane permeability caused by lysoglycerolipids was not intervened in the enhancing effect of lysoglycerolipids. In addition, if the uptake amount was increased by increasing membrane permeability, it should also be increased in dispersed cells, which was not observed. Therefore, that candidate mechanism was dismissed.

Similar to our previous studies on carotenoid uptake [22,23,24,25], the present study also monitored the concentration of residual micellar vitamin D in the medium after incubation with Caco-2 cells because only micellar vitamin D can be taken up by cells. In the experiments with differentiated Caco-2 cells, the residual concentrations of micellar vitamin D or carotenoid tended to be higher in lysoPC mixed micelles than in NoPC mixed micelles. The enhancing effect of lysophosphatidylcholine may be due to the high concentration of residual micellar vitamin D. However, the trend of the results on the uptake of vitamin D by dispersed Caco-2 cells was reversed between NoPC mixed micelles and lysoPC mixed micelles (3 d vs. 7 d, respectively), and the residual concentrations of micellar vitamin D were almost constant (Figure 6c), indicating that the residual concentrations did not influence the enhancing effect of lysophosphatidylcholine under the present experimental conditions.

Vitamin D exerts its function after being metabolically converted into an activated form. In this study, the biological activities of activated vitamin D were estimated by PASS online. Vitamin D originally had been identified as an anti-rickets activity factor. The activities of vitamins D_2_–D_7_ have long been investigated by performing a line test using rats. A comparison of vitamins D provided as a crystal showed that vitamins D_2_ and D_3_ had the highest activities and were similar [109], the activity of vitamin D_4_ ranged from 50–75% of that of vitamin D_2_ [92], and the activity of vitamin D_5_ was very low and 1/180th that of vitamin D_3_ [124]. Studies have also been conducted on UV-irradiated precursors, but not crystals. In this case, the activity may appear to be lower than when using crystals, probably because the conversion rate to the vitamin D form after UV irradiation would not be taken into consideration. The activity of vitamin D_4_ was 1/30th to 1/40th that of vitamin D_2_ [125], the activity of vitamin D_5_ was 1/70th that of vitamin D_2_ [126] or 1/40th that of vitamins D_2_ and D_3_ as UV irradiation precursors [127], and vitamin D_6_ showed little or no activity [126,127,128,129], and the activity of vitamin D_7_ was 1/10th that of vitamin D_2_ [130]. These reports collectively showed that vitamins D_5_–D_7_ had very low anti-rickets activities. However, it should be noted that these activities were limited to anti-rickets activity in rats.

The potencies of 1,25-di(OH)-vitamins D_5_ and D_6_ estimated by PASS online were not very low in terms of bone-related, calcium regulation-related, or vitamin D-like activity because they were significantly different from those assumed from previous reports of anti-rickets activity in rats, as described above. The activity of 1-OH-vitamin D_5_, but not of 1,25-di(OH)-vitamin D_5_, in actual rat studies has been reported [131]. 1-OH-Vitamin D_3_ and 1-OH-vitamin D_2_ were shown to be metabolized into 1,25-di(OH)-vitamin D as an activated form in human liver model cells [132] or in the human body [133]. Therefore, 1-OH-vitamin D_5_ would convert to the activated form in the liver. Although 1-OH-vitamin D_5_ was certainly less active than 1,25-di(OH)-vitamin D_3_ for increasing the blood concentration of calcium in rats, it still showed approximately one-sixth of the activity of 1,25-di(OH)-vitamin D_3_ [131]. The activity of 1-OH-vitamin D_5_ seems to be sufficiently high based on the report above stating that the anti-rickets activity of vitamin D_5_ was 1/70th–1/40th that of vitamins D_3_ or D_2_.

The biological activity of vitamin D may vary between species. In fact, in chicks, the anti-rickets activities were found to be D_3_ > D_4_ > D_2_, an order different from that in rats [109]. There are still many unclear points regarding the difference in anti-rickets activities among vitamins D in other animal species or humans. The strength of biological activities may not always match that of anti-rickets activity. Elucidation of these biological activities deserves future study.

## 5. Conclusions

We investigated the uptakes of six types of vitamins D_2_–D_7_ solubilized in mixed micelles by human intestinal model Caco-2 cells. Vitamins D_2_–D_4_ are commercially available. Vitamins D_5_–D_7_ were obtained by our organic synthesis. The vitamin D uptakes by differentiated Caco-2 cells did not differ markedly among the types of vitamin D except for vitamin D_5_. In other words, the uptake amounts of vitamins D_4_–D_7_ were similar to those of vitamins D_2_ and D_3_, suggesting that taking various types of vitamin D from a wide variety of foods eliminates vitamin D deficiency. Lysophosphatidylcholine enhanced vitamin D uptake, suggesting that the intestinal absorption of vitamin D is enhanced by using phospholipids for cooking or eaten together. Its mechanism involved cell-cell adhesion/cell-matrix adhesion. We also confirmed reduction of cellular cholesterol by micellar lysophosphatidylcholine; in other words, lysophosphatidylcholine showed an enhancing effect on vitamin D uptake by reducing the intercellular barrier formation in Caco-2 cells by reducing cellular cholesterol. In the future, further functional tests and in vivo intestinal absorption tests of vitamins D_5_–D_7_ would be useful. However, it is difficult to conduct such an experiment without synthesizing vitamins D_5_–D_7_ in large quantities. The anti-rickets activities of vitamins D_5_–D_7_ reportedly were lower than those of vitamins D_2_ and D_3_ in rats, but the PASS online estimation suggests that vitamins D other than vitamins D_2_ and D_3_ also have strong biological activities. Verification of the actual effects on the nutritional significance of vitamins D_4_–D_7_, including confirmation of metabolic conversion into activated forms, is a topic for future research.

## Figures and Tables

**Table 1:** The predicted biological activities of 1, 25-di(OH)-vitamin D.

	Vitamin D_2_		Vitamin D_3_		Vitamin D_4_/D_7_^∗^		Vitamin D_5_		Vitamin D_6_	
Activity	Pa ^1^	Pi ^2^	Pa	Pi	Pa	Pi	Pa	Pi	Pa	Pi
Anti-osteoporotic	0.982 ^a^	0.003	0.973 ^b^	0.003	0.969	0.003	0.965	0.003	0.970	0.003
Bone diseases treatment	0.981 ^a^	0.003	0.976 ^b^	0.003	0.968	0.003	0.966	0.003	0.966	0.003
Vitamin	0.977 ^b^	0.000	0.975	0.000	0.936	0.000	0.978 ^a^	0.000	0.976	0.000
Hyperparathyroidism treatment	0.946 ^a^	0.000	0.933 ^b^	0.000	0.883	0.000	0.882	0.000	0.901	0.000
Calcium regulator	0.902 ^a^	0.001	0.876	0.002	0.869	0.002	0.870	0.002	0.880 ^b^	0.001
Vitamin D-like	0.869 ^a^	0.000	0.757	0.000	0.578	0.000	0.791	0.000	0.844 ^b^	0.000
Vitamin D receptor agonist	0.816 ^a^	0.000	0.693	0.000	0.680	0.000	0.686	0.000	0.738 ^b^	0.000
Bone formation stimulant	0.578	0.004	0.598	0.004	0.628 ^b^	0.003	0.634 ^a^	0.003	0.627	0.003

^1,2^ Pa (probability to be active) and Pi (probability to be inactive) values were estimated by PASS (Prediction of Activity Spectra for Substances) online. The closer the Pa value is to 1, the higher the activity. The larger the Pi value, the more inactive it is. ^∗^ These isomers cannot be distinguished by PASS. ^a^: Highest activity among vitamins D. ^b^: Second highest activity among vitamins D. ^a,b^: both judged by both Pa value and Pi value.
